# Primary Leiomyosarcoma of the Ovarian Vein Causing Obstructive Uropathy: A Case Report

**DOI:** 10.7759/cureus.28510

**Published:** 2022-08-28

**Authors:** Fouad Hajji, Mohamed A Azami, Sanaa Adlouni, Abderrazak Benazzouz, Omar Ghoundale

**Affiliations:** 1 Department of Urology, Cadi Ayyad University, Ibn Sina Military Hospital, Marrakech, MAR; 2 Department of Pathology, Cadi Ayyad University, Ibn Sina Military Hospital, Marrakech, MAR; 3 Department of Radiology, The Clinical Marrakech, Marrakech, MAR; 4 Department of Urology, Cadi Ayyad University, Ibn Sina Military Hospital, Marrakesh, MAR

**Keywords:** retroperitoneal sarcoma surgery, obstructive hydronephrosis, ovarian vein, vascular leiomyosarcoma, retroperitoneal sarcomas

## Abstract

Retroperitoneal vascular leiomyosarcoma (RVLMS) are rare soft-tissue sarcomas that most commonly arise from large blood vessels and have a poor prognosis. We present the case of a 61-year-old woman who presented with isolated left flank pain. Abdominal computed tomography and magnetic resonance imaging revealed a 5 cm retroperitoneal soft-tissue mass that was contiguous with the left ovarian vein and connected to the proximal ureter, causing hydronephrosis. As ureteroscopy suggested extrinsic compression of the ureter, a percutaneous biopsy of the mass was obtained, whereupon diagnosis of leiomyosarcoma was made. Radical en-bloc excision of the tumor, including the involved upper urinary tract and the gonadal vein, was performed. The tumor proved to be a leiomyosarcoma arising from the ovarian vein wall. No adjuvant therapy was planned, and no recurrence was noticed at her 24-month follow-up. Primary RVLMS of the ovarian vein is an uncommon condition. To date, only a few sporadic cases have been reported in the literature. What makes the present case further interesting is the unusual tumor’s relationship with the patient’s ureter, raising both diagnostic and management challenges. To our best knowledge, this is so far the fourth reported case of its kind to cause ureteral obstruction.

## Introduction

Leiomyosarcoma (LMS) is a rare malignant mesenchymal tumor of smooth muscle origin. Among adults 55 and older, it is the second most common subtype of soft-tissue sarcoma to affect the retroperitoneum (28%) [1, 2]. Most cases of retroperitoneal LMS are of vascular origin and have a poor prognosis [3]. Retroperitoneal vascular LMS (RVLMS) is uncommon among soft tissue sarcoma (1/100000 malignant tumors), making up only 2% of all cases of LMS [3, 4]. The inferior vena cava (IVC), renal vein, and central adrenal vein are most commonly involved (60%), with clear female predominance (female-to-male ratio of 4:1) [5]. However, RVLMS arising from the ovarian vein is an uncommon condition in clinical practice; only a few cases have been reported in the literature. This report describes an unusual case of LMS of the ovarian vein wall with ureteral obstruction and subsequent hydronephrosis.

## Case presentation

A 61-year-old woman with an unremarkable past medical history presented with intermittent and isolated left flank pain of six months duration. Her physical examination was normal. Abdominal ultrasonography showed left hydronephrosis. Routine blood tests, urinalysis, culture, and cytology were normal.

**Figure 1:**
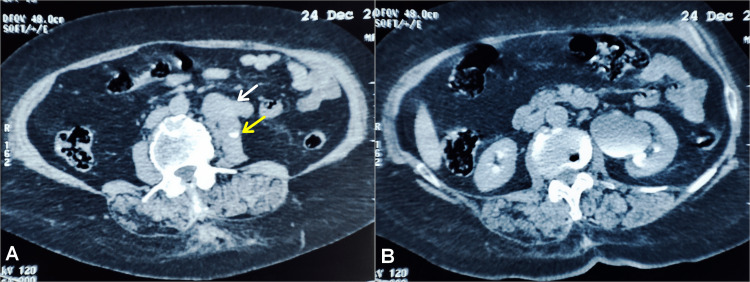
Axial contrast-enhanced CT of the abdomen showing a circumscribed, lobulated, and heterogeneously enhanced left retroperitoneal mass No evidence of necrosis or hemorrhage was observed in the tumor. There were neither calcified areas nor cystic components inside, and no signs of metastatic disease were noticed elsewhere. (A) Axial CT image showing the left ureter (yellow arrow) passing through the dorsal side of the mass (white arrow). (B) Axial CT image showing left hydronephrosis.

Contrast-enhanced computed tomography (CECT) of the abdomen revealed a 5 cm retroperitoneal mass, projecting at the left L3 level and causing obstruction of the proximal ureter. No tissue plan was noticed between the mass and the psoas muscle (Figure 1). Tumor markers, including carcinoembryonic antigen, carbohydrate antigen 19-9, alpha-fetoprotein, beta human chorionic gonadotropin, and lactate dehydrogenase, were within normal limits. Left ureteroscopy showed a narrow proximal-ureteral lumen with intact mucosa and no intraluminal lesions, suggesting extrinsic compression. A double-J stent was then inserted to drain the collecting system.

**Figure 2:**
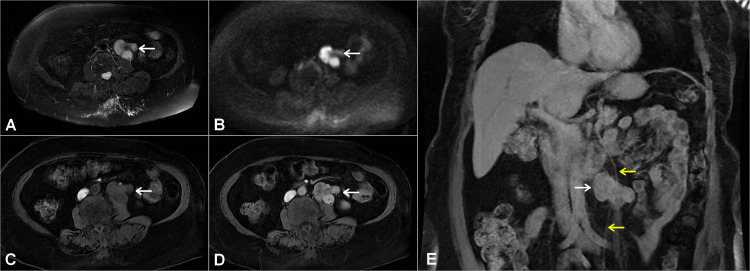
Abdominal MRI image showing a retroperitoneal macroscopic-fat free soft tissue mass that was completely separated from the ipsilateral psoas muscle (white arrows) (A) Axial T2 and (B) diffusion-weighted MRI images showing a mass with high signal intensity. (C) Axial T1-weighted MRI image showing a mass with low signal intensity and (D) heterogeneous gadolinium enhancement. (E) Coronal T1-weighted MRI image showing the left ovarian vein (yellow arrows) running throughout the mass.

**Figure 3:**
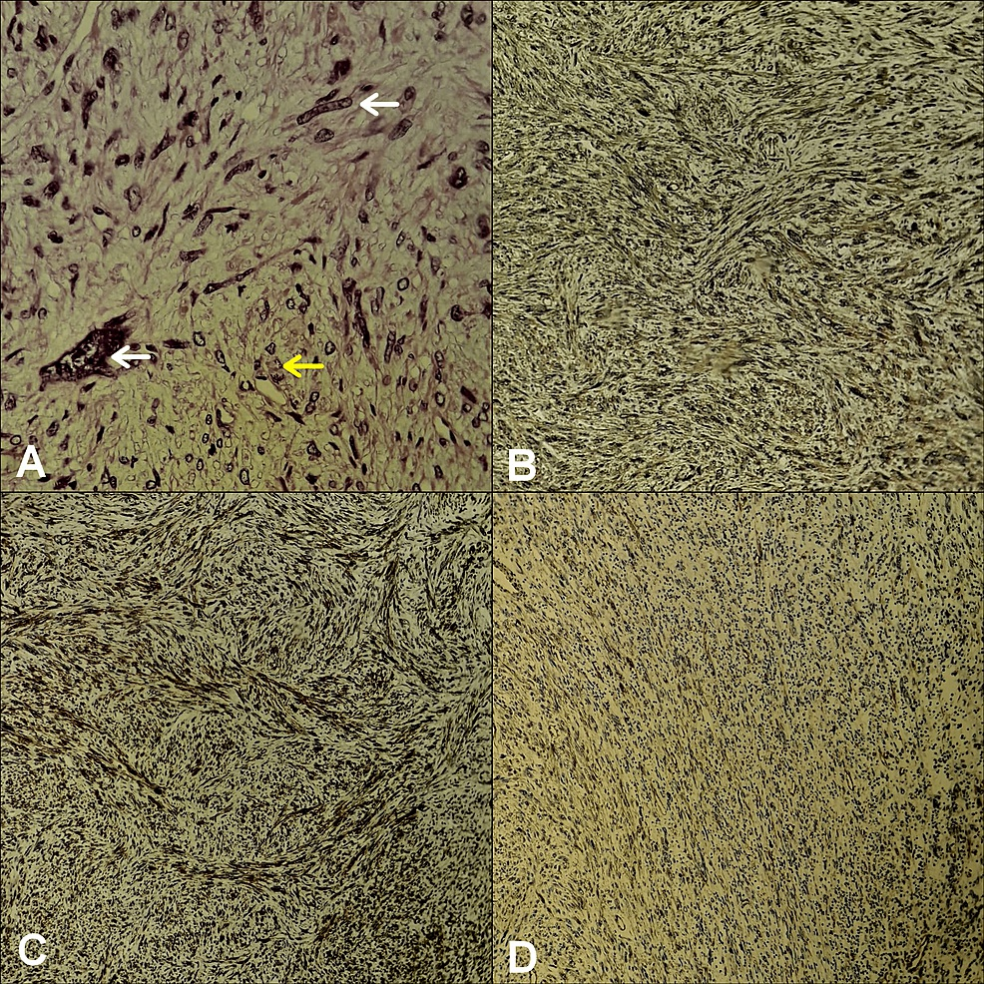
Photomicrograph image showing histological and immunohistochemical features pathognomonic of leiomyosarcoma (A) Hematoxylin and eosin staining (HE, ×200) showing atypical spindle cells arranged in a fascicular pattern with nuclear atypia (white arrows) and frequent mitoses (yellow arrow). Immunohistochemical staining (×100) showing positive results for (B) smooth muscle actin, (C) H-Caldesmonn and (D) Vimentin.

On magnetic resonance imaging (MRI), the mass was completely separated from the psoas muscle by a fat plane, and the left ovarian vein was seen to be running through the mass. However, no abnormal macroscopic fat was detected in the lesion (Figure 2). Consequently, a percutaneous CT-guided retroperitoneal coaxial biopsy was performed. Histopathology and immunohistochemistry findings were consistent with LMS (Figure 3). The patient was told that her tumor most likely originated from the ovarian vein rather than to be of ureteral origin.

**Figure 4:**
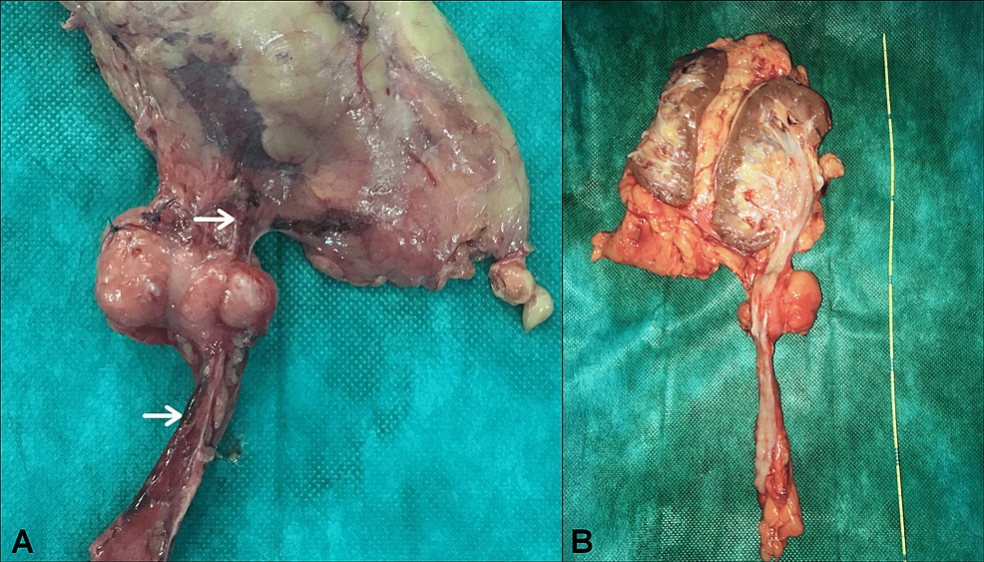
Gross pathological specimen showing a well-defined, encapsulated and firm lobulated solid mass tightly adherent to the proximal ureter (A) The left ovarian vein was found to be passing through the mass (white arrows). (B) Cut section showing an intact ureteral mucosa with no intraluminal lesions.

Exploratory laparotomy revealed a retroperitoneal firm mass located about 1.5 cm beneath the ureteropelvic junction, completely wrapping approximately 4 cm of the proximal ureter and tightly adherent to the ovarian vein. Based on these intraoperative findings, radical en bloc excision of the tumor, including the involved upper urinary tract and the gonadal vein, was performed (Figure 4). The post-operative period was uneventful.

**Figure 5:**
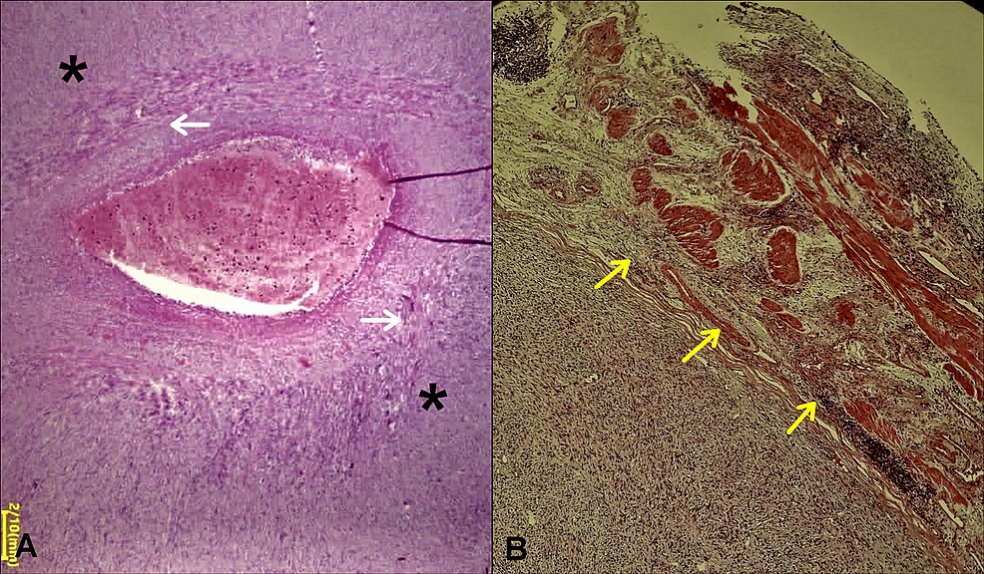
Photomicrograph image showing histopathological features of leiomyosarcoma (HE, x40) (A) The tumor was seen to originate from the media of the ovarian vein wall (white arrows) with extra-luminal extension (*). (B) The tumor invaded the periureteral fat tissue (yellow arrows).

On final histopathology, the tumor proved to be an LMS of the ovarian vein wall, showing extra-luminal extension and invading the adjacent periureteral fat tissue with negative surgical margins (Figure 5). Twelve mitoses per 10 high-power fields were noticed (HPF) with no evidence of necrosis. According to the multidisciplinary cancer team, no adjuvant therapy was required, but regular follow up strongly advised. At her two-year follow-up, she was symptomless and had no evidence of local recurrence nor distant metastasis.

## Discussion

Primary RVLMS of the ovarian vein is an unusual condition; only about 23 cases have been recorded [6-12]. Despite our extensive review of English literature, we could not find any similar case of RVLMS of the gonadal vein in a male patient. Aside from potential hormonal and gynecological factors, the clear female predominance seen in this entity is difficult to explain.

Usually, RVLMS grows slowly to a large size before detection as an incidental finding at an abdominal examination or imaging [2]. When symptomatic, they are often associated with nonspecific complaints caused by displacement and/or compression rather than invasion in neighboring structures. In this case, the RVLMS encased the ureter and invaded its periureteral fat tissue, causing obstructive uropathy. To our best knowledge, so far this is the fourth reported case of LMS in the ovarian vein to cause ureteral obstruction [11, 13, 14].

RVLMS can be detected by contrast-enhanced computed tomography (CECT); it typically appears as a large macroscopic-fat free soft-tissue mass that is separate from retroperitoneal organs and is contiguous with the vascular structure, often with areas of necrosis, hemorrhage, or cystic changes [1, 2]. In this case, the tumor had no characteristic imaging appearances on CT, leading to a diagnostic ureteroscopy to primarily rule out mucosal lesions of the ureter. Consequently, primary soft-tissue retroperitoneal sarcomas, other malignant lesions, metastatic disease, retroperitoneal fibrosis with atypical periureteral location, and benign masses had all been put forward to explain extrinsic compression of the ureter.

Owing to its various pulse sequences and multiplanar capacity [13], MRI was useful to demonstrate the tumor’s relationship with our patient’s psoas muscle and ovarian vein. It was actually helpful in ruling out rhabdomyosarcoma of the psoas muscle as well as in predicting the vascular origin of the mass, which had exhibited signal changes compatible with signal intensities of LMS. Some reports suggest that multidetector CT and three-dimensional angiography are also useful in identifying the vascular origin of such tumors [8, 15]. 

The diagnosis of LMS in our patient, despite a high index of radiologic suspicion, had required a pretreatment biopsy, which had proved to be safe, feasible, and effective using a retroperitoneal route and immunohistochemical staining. Indeed, patients with no history of malignancy and normal biomarkers should have their macroscopic fat-free retroperitoneal mass sampled on a utility-based approach whenever histological findings may influence management [1].

Approximately 60% of RVLMS present an extravascular growth pattern [1, 2]. The present case had shown extraluminal extension leading to invasion of the ureter, which had made differentiation from external compression of the ovarian vein challenging. Besides tunica media of blood vessels, retroperitoneal LMS may originate from any smooth muscle-containing structures of the retroperitoneum, including muscularis of the ureter. Indeed, primary LMSs of the ureter do exist [16]. Had diagnostic ureteroscopy not been performed in this case, our patient would have been easily misdiagnosed as having an LMS of the ureter.

However, the origin of retroperitoneal LMSs may have no clinical relevance in such clinical scenarios as they all have similar treatments. Nonetheless, it is important to distinguish a non-vascular soft-tissue LMS (i.e., LMS of the ureter) from an LMS of vascular origin, as the latter tends to metastasize through hematogenous spread, leading to a worse prognosis [3]. None of the cases of RVLMS of gonadal location reported to date were diagnosed at metastatic stage [6], unlike those of other vascular locations than IVC, in which synchronous metastases are identified in 12% of cases [4].

There are currently no formal guidelines on the management of RVLMS. Nevertheless, complete excision of the tumor offers the best chance of cure; neither neoadjuvant nor adjuvant chemo- and /or radiotherapy have proved to be effective at improving local control and survival [1,2, 5]. However, one or more adjacent organs often need to be removed together with the lesion [1, 2]. The present case, with a small-sized tumor, could have been managed conservatively. Albeit technically challenging, segmental ureterectomy can be preferred over tumorectomy, but potential risk factors include positive surgical margins and recurrence. Consequently, radical en-bloc excision of the tumor and the ovarian vein with nephroureterectomy is a good treatment option for this condition, especially in patients with preserved renal function and normal contralateral upper urinary tract.

Tumor size, depth of invasion, differentiation, mitosis count, and microscopic necrosis have been described as prognostic factors of vascular LMS [3]. Besides invasion of the ureter, the present case had proved to be of good prognosis, especially after achieving complete resection with clear surgical margins. However, long-term follow-up was strongly advised as local recurrence and/or distant metastasis may occur years after surgery [6].

## Conclusions

In the setting of an indeterminate retroperitoneal mass, RVLMS should be considered if the lesion is contiguous with a vessel. Multisequence and multiplanar MRI may be of great help in predicting the histologic subtype and the vascular origin of such neoplasms. However, the ovarian vein is a rare and unusual location of the LMS, and cases of gonadal vein LMS causing obstructive uropathy are even rarest. Hence, pretreatment ureteroscopy and biopsy should be obtained. Conservative surgery can be used in selected cases. However, radical en-bloc excision of the tumor and the ipsilateral upper urinary tract is the standard treatment option in our patient with ureteral invasion.
